# Comparison of Chicken Immune Responses to Immunization with Vaccine La Sota or ZG1999HDS Strain of Newcastle Disease Virus

**DOI:** 10.3390/life12010072

**Published:** 2022-01-05

**Authors:** Gordana Nedeljković, Hrvoje Mazija, Željko Cvetić, Mladen Jergović, Krešo Bendelja, Željko Gottstein

**Affiliations:** 1 Veterinary and Food Safety Directorate General, Ministry of Agriculture, 10 000 Zagreb, Croatia; 2 Faculty of Veterinary Medicine, University of Zagreb, 10 000 Zagreb, Croatia; mazija@vef.unizg.hr; 3 Laboratory of Immunology, Centre for Research and Knowledge Transfer in Biotechnology, University of Zagreb, 10 000 Zagreb, Croatia; zeljko.cvetic@unizg.hr (Ž.C.); kreso.bendelja@unizg.hr (K.B.); 4 Department of Immunobiology, The University of Arizona College of Medicine, Tucson, AZ 85719, USA; mjergovic@email.arizona.edu; 5 Department of Poultry Diseases with Clinic, Faculty of Veterinary Medicine, University of Zagreb, 10 000 Zagreb, Croatia

**Keywords:** Newcastle disease, ZG1999HDS strain, chicken, immunization, antibody response, cell-mediated immunity

## Abstract

Newcastle disease (ND) is a highly contagious avian disease. Global control of ND is mainly based on vaccination of poultry; however, reported outbreaks of ND in vaccinated flocks indicate a constant need to re-evaluate the existing vaccines and a development of the new ones. In this study, 4-week-old male chickens of the layer commercial hybrid were immunized oculonasally with a commercial NDV live La Sota vaccine (LS group), a suspension of lyophilized NDV strain ZG1999HDS (ZG group), or saline (Control (K) group). Antibody response was determined by haemagglutination inhibition (HI) assay. Cell-mediated immunity (CMI) was characterized by immunophenotyping of leukocyte’s and T-lymphocyte’s subpopulations (flow cytometry). Applied NDV strains did not cause any adverse reaction in treated chickens. Both strains induced the significantly higher HI antibody response in comparison to the control group, and overall antibody titer was higher in ZG group than in LS group. CMI, manifested as a higher proliferation of B- and T-helper cells, yielded better results in the ZG groups than in the LS group. Based on the obtained results, we conclude that the strain ZG1999HDS is immunogenic and is a suitable candidate for further research and development of poultry vaccines.

## 1. Introduction

Newcastle disease (ND) is, along with avian influenza, the most significant disease of poultry, posing an enormous economic burden for the global poultry industry. ND is caused by Avian orthoavulavirus-1 (AoAvV-1), historically known as Newcastle disease virus (NDV) [1], a member of the order *Mononegavirales*, family *Paramyxoviridae*, and genus Orthoavulavirus [2]. Depending on the species, age, and immunity status of the host and the strain of AoAvV-1, the clinical manifestation of ND varies, from unapparent infection to peracute infection with 100% mortality [3].

Global control of ND is, in addition to non-specific measures of good management practice, sanitation, and biosecurity, often based on specific immunoprophylaxis, i.e., vaccination. For this purpose, live vaccines containing apatogenic, lentogenic, or asymptomatic intestinal NDV strains, or inactivated vaccines are used [3,4,5]. Although currently available commercial vaccines prevent the morbidity and mortality of ND, they are unable to prevent infection with, replication, and shedding of virulent NDV strains [6,7,8]. Furthermore, prevalent contemporary circulating velogenic NDV strains are viscerotropic in nature, which makes widely used commercial vaccines based on pneumotropic strains inadequate to protect animals. This is evidenced by the outbreaks of ND in vaccinated flocks with great economic losses, e.g., Egypt in 2005, and more recently in India in 2015–2016 [9]. Moreover, due to the lentogenic or apatogenic nature of vaccine strain, the vaccine failure can be attributed to the predominant use of vaccines of genotype (gt) 1 (VG/GA, I-2) or gt 2 (LaSota, Hitchner B1), which are genetically heterologous to predominantly circulating virulent strains of gt 7.

Haemagglutination inhibition (HI) assay or enzyme-linked immunosorbent assay (ELISA) are commonly used 3 to 4 weeks post vaccination for assessment of the immune status of animal or flock [10,11,12,13]. Antibodies appear in serum and mucosal membranes 4 to 10 days post immunization (dpi) [13] and reach peak levels at two to three weeks p.i. [12,14].

In addition to the development of antibodies against NDV, cell-mediated immunity (CMI) plays an important role in protection against ND. It can be detected as early as two to three dpi or after field infection. CMI can be characterized by immunophenotyping, i.e., determination of the total leukocytes number and individual subpopulations of leukocytes (monocytes, T- and B-cells) in the peripheral blood (i.e., peripheral blood mononuclear cells, PBMCs) [15,16], but also by in vitro tests of their functional capacity.

Flow cytometry is an objective analytical method that allows for qualitative and quantitative determination of biological and phenotypic characteristics of cells. Immunophenotyping uses monoclonal antibodies specific for distinctive antigens present on cells, i.e., cluster of differentiation (CD), and could be applied for identification and characterization of different immune cell subpopulations. Thus, CMI can be measured by the proliferation of lymphocytes (i.e., rise in number) and identification of alternations in lymphocyte subpopulations in peripheral blood.

ZG1999HDS strain of NDV was isolated from an outbreak in July of 1999 at a broiler farm. Previous studies on the NDV strain ZG1999HDS confirmed immunogenicity [17,18] lentogenic nature [19,20,21], and cytolytic (oncolytic) activity in cell culture [22].

In this study, ZG1999HDS strain was further assessed for immunogenic properties, in comparison to a commercially available vaccine of La Sota strain. The comparison between the extent of CMI induction alongside the antibody response elicited by ZG1999HDS strain and the ones elicited by La Sota vaccine strain provided means for evaluation of ZG1999HDS strain as a prospective candidate for further research and vaccine development.

## 2. Materials and Methods

### 2.1. Trial Animals

The study animals included a total of 150 male chickens of the commercial layer hybrid TETRA-SL LL (“long life”) (Bábolna Tetra Ltd., Bábolna, Hungary). One-day-old chickens were obtained from the Valipile hatchery (Sesvetski Kraljevec, Croatia) and moved into the experimental establishment at the Department for Poultry Diseases with Clinic, Faculty of Veterinary Medicine, Zagreb. Chickens were placed in cages with ad libitum supply of water and feed (commercial feed mixture for the respective poultry category). Chickens were kept in a single room until the onset of the trial on the 28th day; meanwhile, the weaning of maternally-derived antibodies (MDA) specific for NDV was determined in the randomly selected 10 chickens in weekly intervals. After the MDA weaning, 28-days-old chickens were divided into three groups (ZG, LS, and K) with 50 chicks in each and moved to cages in separate rooms.

During the trial, the overall health status of the chickens was monitored by the inspection during daily handling, feeding, and cleaning, and, if necessary, by other methods of clinical examination. The microclimatic housing conditions during the study were set in accordance with the hybrid manufacturer’s instructions.

All the required permits for performing experiments on animals were obtained prior to the onset of the trial from the relevant competent authorities. For the purpose of animal sacrifice, the 80% CO_2_ mixture was used in compliance with all prescribed measures for the protection of animals in the experiment.

### 2.2. Viral Preparations

NDV strain ZG1999HDS was isolated from the outbreak in July of 1999 at a broiler farm in northwest part of Croatia. The outbreak took the toll of 77% mortality of the affected flock over the 17 days. Initially, the existence of viral or bacterial co infection, in addition to unfavorable environmental conditions, was considered as a cause of established mortality. In addition, negative serology findings at the time strongly suggested that the broiler flock was not vaccinated against infectious bronchitis, infectious bursal disease, or Newcastle Disease. Bacteriology further excluded salmonellosis, and feed mixture fed to chickens did not contain mycotoxins T-2 or DAS.

The virus was isolated on chicken embryos from the lung tissue, but not the brain, of broiler chickens that died at the age of 28 days, thus suggesting it is pneumotropic in nature. NDV was further confirmed, and avian influenza virus was excluded in neutralization test with respective hyperimmune sera. Initial pathogenicity parameters were mean death time of 9–10 days old chicken embryos (MDT) >120 h (5–6 days) and hemagglutination (HA) titer of 1:64 (1:2^6^). Passage on chicken SPF embryos resulted in viral allantoic fluid (VAF) that was lyophilized (i.e., freeze-dried). The determined HA titer for VAF used in the study was 1:256 (1:2^8^). Current HA titer of 1:2048 suggests non-avian origin of virus and points to pigs being kept at the same holding of outbreak as the possible source of virus.

For comparison, we have used commercial vaccine against ND (Pestikal^®^ La Sota SPF, Genera, Rakov potok, Croatia). Based on the data provided by the manufacturer [23], the values of pathogenicity indices were as follows: ICPI = 0.18, IVPI = 0.0, and MDT >103 h. Since the acquisition of Genera company, the vaccine seed virus is currently commercially available under the name Avishield^®^ ND (Dechra, UK).

Both NDV strains belong to Class II and genotype II. Deduced amino acid sequence of the F0 cleavage site for both strains was ^112^GRQGR↕L^117^, a motif corresponding to lentogenic strains. However, the nucleotide identity, compared to variable region of F gene, was 95.9% between the two strains (unpublished data).

### 2.3. Animal Procedures

Immunization of chickens in the trial groups was performed using a La Sota vaccine at a dose >10^6.0–7.0^ EID_50_/chick (LS group) or by suspension of a lyophilized VAF containing strain ZG1999HDS of NDV at a dose of ~10^6.5^ EID_50_/chick (ZG group). The VAF and the vaccine were dissolved in sterile saline immediately prior to use. Virus preparations were applied oculonasally, a drop of 0.02 mL in the left eye and a drop of 0.02 mL in the left nostril, in an attempt to mimic the natural route of infection. Chickens in the control group (K group) were treated in the same way with the same amount of sterile saline.

Sampling of animals was performed on five randomly selected chickens per group.

Blood collection from jugular vein was performed immediately before animal sacrifice for the collection of spleen samples (not shown here):

(1) for hematology differentiation (data not shown here) and flow cytometry: with the addition of anticoagulant (Heparin, PLIVA d. d., Zagreb, Croatia) in time intervals prior to immunization (0), 3, 5, 7 and 14 dpi. Samples for flow cytometry were analyzed immediately after the blood collection.

(2) for the HI assay (10 samples/group = from randomly selected 5 chickens to be sacrificed and additional 5 chickens that were returned to the cages): without anticoagulant at time intervals 0 (before immunization), 7, and 14 dpi. Sera were separated from the whole blood and stored at −20 °C until analysis.

### 2.4. Laboratory Procedures

#### 2.4.1. Determination of Humoral Immunity by Haemagglutination Inhibition (HI) Assay

HI assay initially served to monitor the weaning of maternally derived antibodies (MDA) for NDV and thus assess the risk of MDA interference with viruses used to immunize chickens.

During the trial, the adaptive antibody immune response was determined by NDV-specific HI antibody titer in sera. The HI assay was performed in 96-well U-bottom microtiter plates by the standard beta procedure [24], as prescribed in the standard operating procedure (SOP) of reference laboratories [25], with a modification that the VG/GA vaccine strain (Avinew, Merial, Lyon, France) was used as an antigen.

#### 2.4.2. Determination of Cell-Mediated Immunity by Differentiation of the Total Number of Chicken Leukocytes and Their Subpopulations in Flow Cytometry

The procedure of leukocyte isolation and flow cytometry was performed at the Department for Cellular Immunity, Institute of Immunology in Zagreb.

Leukocytes (i.e., mononuclear cells in peripheral blood of chickens, chPBMCs) were isolated from heparinized whole blood of chicken by modified ficoll-density gradient procedure, as previously described [26]. In short, heparinized chicken whole-blood samples were divided into two tubes (polystyrene, 4 mL, BD Biosciences) with 1 mL in each tube and diluted 1:1 with phosphate-buffered saline (PBS, pH = 7.2−7.4, Institute of Immunology, Zagreb, Croatia). Modification of extraction method wasin additional treatment of heparinized blood with 3% dextran T-500 (GE Healthcare Bio-Science, Uppsala, Sweden) prior to the treatment with ficoll (Histopaque^®^-1077, Sigma-Aldrich Chemie, Steinheim, Germany).

Cell staining and immunophenotyping were performed as previously described [26]. In short, the concentration of cells was adjusted to 250,000 cells/mL in two separate 4 mL tubes designated as L- and T-tubes. Cells in each tube were stained with different mouse monoclonal antibodies (MAbs) for chicken leukocyte surface antigens, i.e., CDs (SouthernBiotech, Birmingham, AL, USA), conjugated with fluorescent dyes (Table 1), as set for T- and L-panel (see below). Unlabeled (UNLB) mouse monoclonal antibody for chicken γδ- TCR surface marker was stained with secondary antibodies for murine IgG antibodies from Zenon^®^ Mouse IgG Labelling Kit Alexa Fluor 647 (Molecular Probe, Eugene, OR, USA). The minimum number of monoclonal antibodies to be added was previously determined by titration.

The following two MAbs panels were used for multicolor labeling of chPBMCs:

Leukocyte panel (L-panel): MAbs -CD45-APC, -Mo/mf-R-PE, Bu-1-FITC CD3- SPRD; and T-cell panel (T-panel): MAbs -CD3- SPRD, -CD8α-FITC, -CD4-R-PE, and -γδTCR (TCR1)-UNLB + secondary anti-mouse Alexa Fluor 647 (APC).

Labelled cells were fixed with fixation buffer (Institute of Immunology, Zagreb, Croatia; composition: 2% formaldehyde in Dulbecco’s phosphate buffer (DPBS)) prior to the acquisition of at least 20,000 events, with size and granularity matching mononuclear cells i.e., leucocytes for L panel or lymphocytes for T-panel, on LSRII flow cytometer (Becton Dickinson, Mountain View, CA, USA).

Multiparametric data analysis to determine the frequency of individual subpopulations of leukocytes and T-cell was performed using FlowJo software (Version 7.6.5, Tree Star, Inc., Ashland, OR, USA). The gating strategies specific to L-panel and T-panel are shown in Figures S1 and S2 in Supplementary Materials.

### 2.5. Statistical Data Analysis

The results were analyzed with STATISTICA 12 (StatSoft Inc., OK, USA, 2013). Basic data analysis used the methods of descriptive statistics. The normality of the data distribution was verified by the Kolmogorov–Smirnov test. Given the normality of the distribution of results, the differences between the experimental and control groups were analyzed using the one-way ANOVA or Kruskal–Wallis method.

## 3. Results

### 3.1. Overall Animal Health in the Experiment

The health status of the chickens during the trial was monitored daily by inspection during routine animal handling, feeding, and cleaning. No adverse reactions or death of the chickens in connection to the applied viruses were observed during the entire trial.

### 3.2. Humoral Immunity

#### 3.2.1. Weaning of MDA

**Figure 1:**
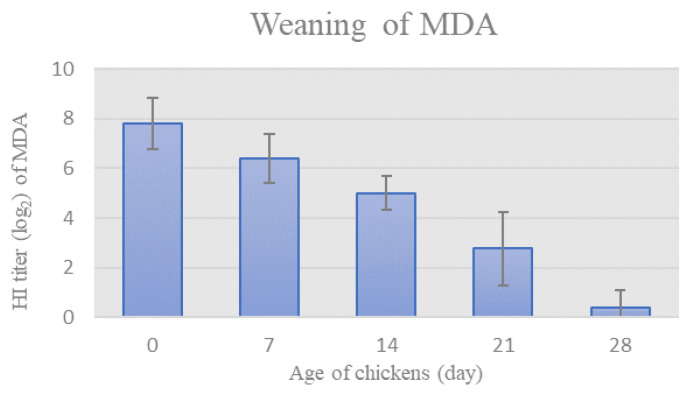
Weaning of MDA, as determined weekly by HI titer (Mean ± SD).

Prior to the onset of the trial, HI assay was used to monitor the weaning of NDV-specific MDA in chickens’ sera. The mean HI titer of MDA specific for NDV gradually decreased in chicken sera in weekly intervals, as seen in Figure 1. The HI titer of MDA reached values below the protective level on day 28th (HI titer = 0.4 ± 0.7).

#### 3.2.2. Acquired HI Antibodies

**Figure 2:**
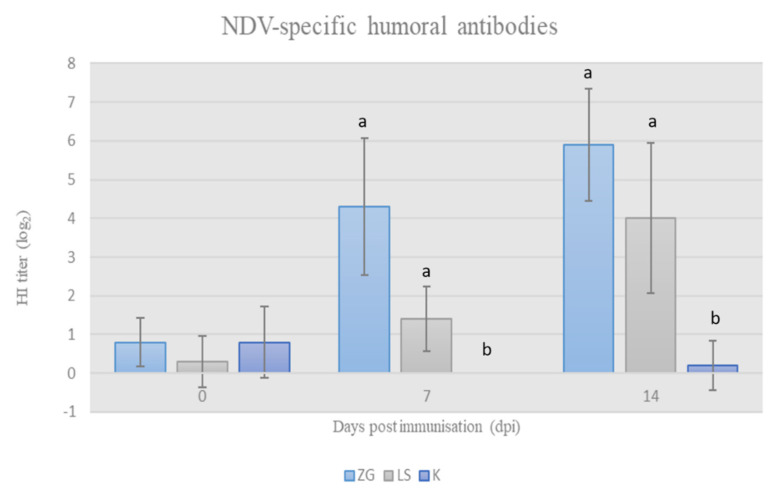
NDV-specific HI antibody titer in chicken sera at weekly intervals following immunisation at 28-days of age (mean ± SD). Statistically significant difference (*p* < 0.05) among the groups is designated with different letters (a, b).

The HI titer of acquired specific antibodies after immunization with tested NDV strains was determined by HI assay. The results of HI titers are presented in Figure 2 and show that the applied viral preparations stimulated the development of specific antibodies in chickens in both trial groups. The increase in antibody titer was statistically significant (*p* < 0.05) in both groups of immunized chickens in comparison to the control group on 7 dpi and 14 dpi, and, simultaneously, the titer of NDV-specific antibodies was higher in the ZG group compared to the LS group.

### 3.3. Cell-Mediated Immunity

#### 3.3.1. Immunophenotyping of Chicken PBMCs

##### Leukocyte-Panel (L-Panel)

**Figure 3:**
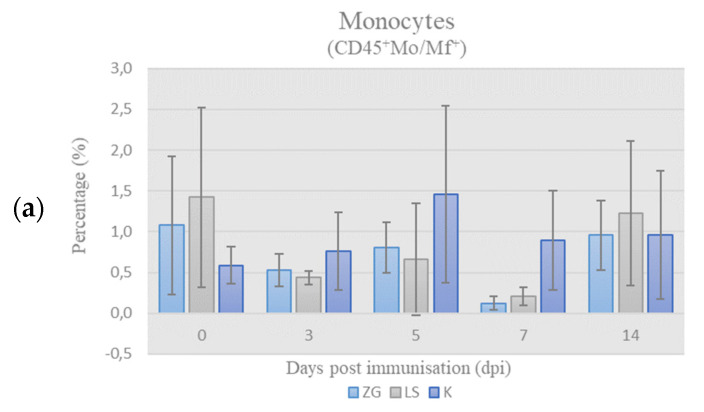

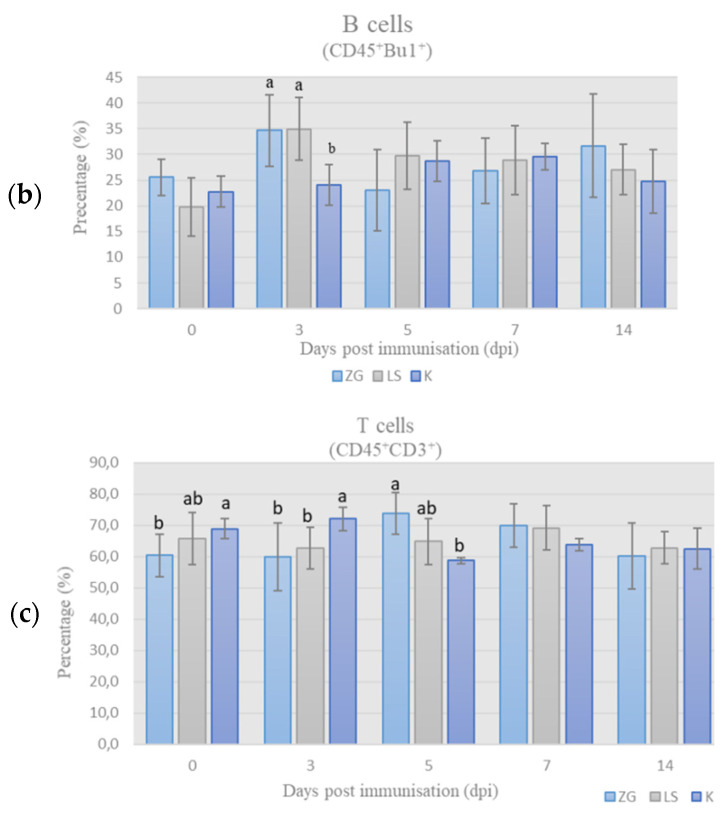
Results of immunophenotyping in L-panel: the percentage (%) of monocytes (**a**), B cells (**b**), and T cells (**c**) in chicken PBMCs on the day of sampling (mean ± SD). Statistically significant difference (*p* < 0.05) among the groups is designated with different letters (a, b, and ab).

Quantitative relationships of chicken PBMC subpopulations (monocytes, B- and T-cells) were presented as a relative ratio, i.e., percentage (%) of these populations in the chPBMCs (CD45^+^ cells = 100%). The results of immunophenotyping of chicken PBMCs labeled according to leukocyte panel (L-panel) are shown in Figure 3.

Monocytes. The results of the frequency of monocytes (CD45^+^Mo/Mf^+^) in chPBMCs are shown in Figure 3a. The values in all groups were below the physiological range, and no significant differences were found during the trial.

Lymphocytes’ subpopulations, B- & T-cells. The results of B-cells’ (CD45^+^ Bu-1^+^) and T-cells (CD45^+^CD3^+^) frequency among chPBMCs during trial are shown in Figure 3b,c, respectively. By 3 dpi, the frequency of B-cells increased and was significantly higher, whereas frequency of the T-cells decreased and was significantly lower in both immunized groups compared to the control group. On 5 dpi, frequency of B-cells decreased, especially in the ZG group, while in the LS group it was still higher than in controls. At the same time frequency, T-cells increased and were significantly higher in ZG group in comparison to LS group and in LS group when compared to K group. Despite the increase in frequency of B-cells by the 7th dpi, the frequency in immunized groups were lower than in control, while the reverse was found for T-cells. At the end of the experiment on day 14, the frequency of B-cells was highest in the ZG, lower in LS, and lowest in control group, but without significant differences. Simultaneously, frequency of T-cells decreased in immunized groups and was lower in ZG group and slightly higher in LS group than in control.

##### T-Lymphocytes’ Panel (T-Panel)

**Figure 4:**
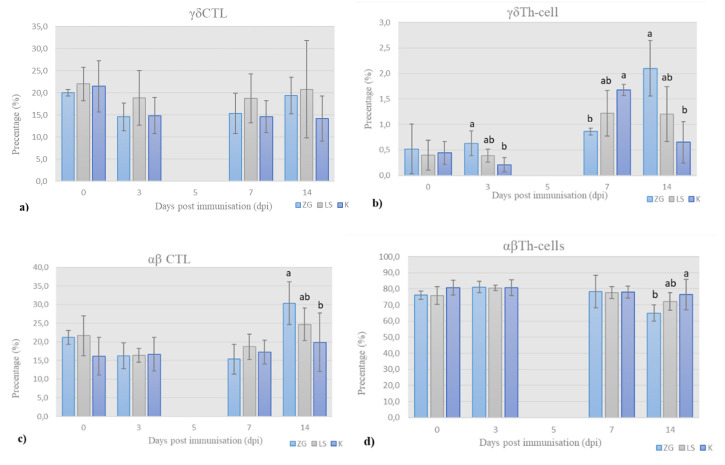
Results of immunophenotyping in T-panel: the frequency (%) of (**a**) γδ cytotoxic T-cells (CTL), (**b**) γδ helper T-cells (Th-cells), (**c**) αβ cytotoxic T-cells (CTL), and (**d**) αβ helper T-cells (Th-cells) in total chicken T-cells, on the day of sampling (mean ± SD). Statistically significant difference (*p* < 0.05) among the groups is designated with different letters (a, b, and ab).

Quantitative relationships of T-cell subpopualtions (γδTCR^+^ T-cells and αβTCR^+^ T-cells, and helper (CD4^+^) and cytotoxic (CD8^+^) T-cells, CTL) in the peripheral blood of chickens were shown as the relative frequency (%) of these populations in total T-cells (CD3^+^ cells = 100). For this purpose, chPBMCs were stained with the cocktail of Mabs in T-panel, and results of flow cytometry analysis are presented in Figure 4. The results for 5dpi are not presented due to staining failure. Gating strategy is presented in Figure S2 in Supplementary Materials.

γδ cytotoxic T-cells (CTL). The prevalence of γδ cytotoxic T-cells, i.e., CTL (CD3^+^TCR1^+^CD8^+^), is shown in Figure 4a. Initial prevalence of γδ CTL decreased by 3 dpi in all the groups and then increased continuously until the end of trial in both immunized groups, and more in LS group. Simultaneously, prevalence of γδ CTL in control group, after initial decrease by 3 dpi, remains almost unchanged until the end of trial.

γδ helper T-cells (Th-cells). The results for prevalence of γδ helper Th-cells (CD3^+^TCR1^+^CD4^+^) are shown in Figure 4b. The prevalence is at low detectable level with values ranging from 0.5 up to 2.1% across the groups. Nevertheless, significant differences were detected. In the ZG group, it increased continuously until the end of trial, when it was significantly different than in LS and K group.

On 3 dpi, the relative proportion of γδ Th-cells was higher in the immunized groups than in the control group, the highest in the ZG group, and significantly different from the control group, while the LS group did not differ significantly from these groups. By 7 dpi, prevalence of γδ Th-cells increased in all the groups, with the highest values in the control group, which was significantly higher than the immunized ZG group but not higher than the LS group. In the second week of the trial, prevalence decreased in control group, was almost unchanged in LS group, and increased in ZG group, again significantly in comparison to control.

αβ Tc-lymphocytes. The prevalence of αβ CTL (CD3^+^TCR1^-^CD8^+^) is shown in Figure 4c. By the 3dpi, the frequency of αβ CTL decreased in the immunized groups and was almost the same in all the all groups. By the end of 1st week, it slightly increased in all the groups. By the end of the trial, prevalence increased in both immunized groups and was significantly higher in ZG than the LS group and in LS group compared to the control.

αβ Th-cells. The prevalence of αβ Th-cells (CD3^+^TCR1^-^CD4^+^) is shown in Figure 4d. This subpopulation was predominant in samples of all the groups. The prevalence of αβ Th-cells increased in immunized groups by 3 dpi and remained almost the same in all the groups during the first week. By the end of trial, it decreased in immunized groups, and difference was significant between control and LS group, and between LS group and ZG group.

## 4. Discussion

Newcastle disease presents a permanent threat to global poultry production, with significant economic impact. Health of animals is pivotal for successful poultry production; thus, non-specific measures and specific immunoprophylaxis, i.e., vaccination, are essential parts of poultry management programs. Vaccination is routinely used in poultry flocks of all ages and production categories. Depending on the type of production, single-dose or repeated vaccination is performed to attain the lasting immunity of birds over production period. Vaccine-induced immunity successfully protects animals from clinical disease and death but is unable to prevent infection with, or replication and shedding of, virulent field strains or challenge viruses.

Vaccination of parental flocks elicits active immune response, and the developed antibodies are transmitted to the offspring, providing adequate protection in the first weeks of life and clear out by the age of 3 to 4 weeks [13,27]. The existence of this passive immunity results in the interference of maternally derived antibodies (MDA) in the serum of chickens with the applied vaccine [27]. To avoid this, live vaccines are administered to chickens via the mucosal membrane of digestive or respiratory tract, thus primarily stimulating local immune response at the site of application and subsequently systemic response [28].

Male chickens of a commercial laying hybrid were used in this study. As anticipated, the chickens were protected with NDV-specific MDA, as indicated by the protective HI antibody titer on the first day (Figure 1). NDV-specific MDA in commercial laying hens disappear within 5 weeks and largely prevent the development of systemic immunity but not the local [27]. In order to characterize the effect of NDV strain ZG1999HDS on the immune response of immunologically naïve chickens, as well as the sequence of events at the initial outbreak, the level of MDA in chicken sera had to be below protective. Therefore, the weaning of MDA was monitored in chicken sera at weekly intervals in order to determine the appropriate time-point to start the experiment. Based on the results (Figure 1), the onset of the trial was set at 28 days when the level of detected antibodies was below protective. The preparations of NDV strain ZG1999HDS or vaccine strain La Sota were applied oculonasally to the 28-days old chickens of the experimental groups, thus imitating the natural route of infection, and saline was applied to the control group via the same route.

Live vaccines containing La Sota strain, when applied via the respiratory tract in newly hatched chickens, can cause adverse reaction manifested in form of damage to the epithelium in the trachea and the disappearance of cilia and thus create a predisposition to secondary bacterial infections [13]. However, for the entire duration of the trial we did not record any adverse reactions or deaths in any of immunized chickens. This finding could be due to the age of trial animals at the time of immunization, as by that age the immune system was fully competent to mount an adequate response. This could also be due to the lentogenic nature of both strains used, as confirmed by the standard in vivo pathogenicity test, and the analysis of the deduced amino acid sequence at the fusion protein (F0) cleavage site. Additionally, such a finding suggests that the significant mortality of broilers during the initial outbreak, when the ZG1999HDS strain was isolated, was unlikely caused by infection with NDV. However, as a pneumotropic agent, it could have contributed to unfavorable effect of environmental and housing conditions. This is supported by its isolation from the lungs but not the brain of the dead chickens, and further by the necropsy findings of an extensive parenchymal hemorrhage in the lungs with effusion of blood into the air sacs, which is indicative of suffocation [22].

Overall immunity, elicited by field infection or vaccination, arises through the process of coordination and interaction of different types of immune cells. Specific humoral and cell-mediated immune responses are governed by the components of innate immunity. With this in mind, we decided to further investigate the cell-mediated immune response in chickens after immunization with ZG1999HDS strain.

The results of this research further supplement previous findings on the immunogenicity of the ZG1999HDS strain. Strain ZG1999HDS strongly stimulated the development of antibodies in comparison to the vaccine La Sota strain, which was manifested by a higher HI antibody titer on the 7 and 14 dpi. In both immunized groups, HI antibody titers were significantly higher than in the control group (Figure 2).

Cell-mediated immunity (CMI) plays a key role in the protection against NDV, as evidenced by the protection of bursectomised (i.e., B-cell depleted) chickens [29]. The CMI to NDV can be characterized by immunophenotyping by flow cytometry, i.e., determining the frequency of effector immune cells in the blood, thymus, and spleen, and other lymphatic organs [16,30]. Immunophenotyping of chicken PBMCs provides good insight into the proliferation of immune cells in response to vaccination or natural infection. Thanks to the growing number of currently available monoclonal antibodies, its use increases, however, due to high costs, it remains limited mostly to research and development of vaccine [15,31].

In this study, the frequency of B-cells (CD45^+^Bu-1^+^) in the population of chicken PBMCs in both immunized groups increased, reaching peak values on 3 dpi (Figure 3b). This finding suggests enhanced activation and proliferation/recruitment of B-cells, and indirect activation of the CMI [32,33], since B-cells also serve as antigen-presenting cells for Th-cells.

The frequency of T-cells followed the increase in B-cells’ percentage; however, it was slightly delayed, reaching “peak” on 5 dpi in both immunized groups and was significantly higher in ZG than in the control groups (Figure 3c). This indicates ongoing humoral immune response induced by both ZG1999HDS and La Sota strains, and is supported by the active role of Th-cells to trigger an appropriate immune response [34,35]. In particular, observed increase on 3 dpi in γδ Th-cells and B cells percentage, along with the high frequency of αβTh-cells (Figure 3a), was probably associated with higher HI titers on day 7 in immunized groups.

Contrary to Th-cells, the increase in CTL frequencies, particularly αβCTL, was delayed and significantly different 14 dpi in both immunized groups. It was established that effectors’ antiviral CTL differentiation follows an initial antibody immune response and is detectable a few days later [32]. This is also the case in other viral infections, e.g., influenza A viral infection [36].

## 5. Conclusions

Based on the obtained results, it can be concluded that the immunization of chickens with ZG1999HDS strain has elicited CMI, which manifested as stronger activation of-cells than the La Sota vaccine strain and as higher frequencies of Th- cells.

Given the stimulating effect of the strain ZG1999HDS on both the antibody and cellular immune response in chickens, it is justified to consider ZG1999HDS strain for further in-depth study and possible development of a vaccine for domestic poultry. This should include analysis of functional capacity of specific immune cells isolated from spleen and blood upon virus-specific stimulation. Finally, to confirm efficacy, a challenge infection with a virulent NDV strain should be performed to provide valuable insights into the protective level of the mounted immunity in chickens.

## Figures and Tables

**Table 1:** Monoclonal mouse-antichicken antibodies (SouthernBiotech, Birmingham, AL, USA), used for multicoloured immunophenotyping in flow cytometry.

Leucocyte Marker (CD)	Clone	Fluorescent Dye	Isotype	Cell Population with Marker
CD45	LT-40	APC	Mouse IgM_κ_	All leucocytes
Mo/Mf	KUL-01	R-PE	Mouse IgG1_κ_	Macrophages and monocytes
Bu-1	AV 20	FITC	Mouse IgG1_κ_	B-cells, Bursal cells
CD3	CT-3	SPRD	Mouse IgG1_κ_	T cells
CD4	CT-4	R-PE	Mouse IgG1_κ_	T helper (Th) cells
CD8α	EP-72	FITC	Mouse IgG2b_κ_	T cytotoxic (Tc) cells
γδ TCR	TCR 1	UNLB(APC)	Mouse gG1_κ_	T cells with γδ surface receptor

## Data Availability

Not applicable.

## Supplementary Materials

The following are available online at https://www.mdpi.com/article/10.3390/life12010072/s1, Figure S1: Gating strategy for L-panel. Figure S2: Gating strategy for T-panel.
