# Supplementary material for: Comparison of Chicken Immune Responses to Immunization with Vaccine La Sota or ZG1999HDS Strain of Newcastle Disease Virus
Source: Life (Basel). 2022 Jan 5;12(1):72. doi: 10.3390/life12010072 (PMC8778274; doi:10.3390/life12010072)
Supplement: Supplementary file 1 [file life-12-00072-s001.zip › life-1454362-supplementary.pdf]

life

MDPI

Supplementary material of Comparison of Chicken Immune Responses to Immunization with Vaccine La Sota or ZG1999HDS Strain of Newcastle Disease Virus

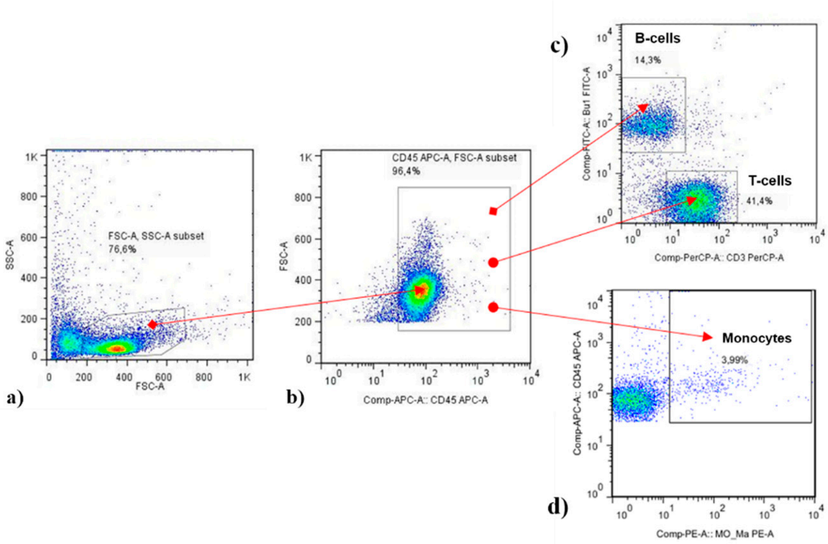

Figure S1. Gating strategy for L-panel. Representative gating strategy for immunophenotyping of chicken PBMC Doublets, debris, and dead cells were excluded from total cells according to L-panel (see in Materials and methods) from mononuclear cells acquired by BD LSRII cytometer. Based on cell size and granularity, gate was placed around mononuclear corresponding– cells, i.e. PBMCs (**Figure S1 a**) and they were additionally confirmed as CD45^{+} cells (leucocytes) (**Figure S1 b**). Mononuclear leukocytes were further differentiated based on the specific markers expression: Bu-1 for B-cells and Mo/Mf for monocytes (**Figure S1.c**), and CD3 for T-cells (**Figure S1.d**).

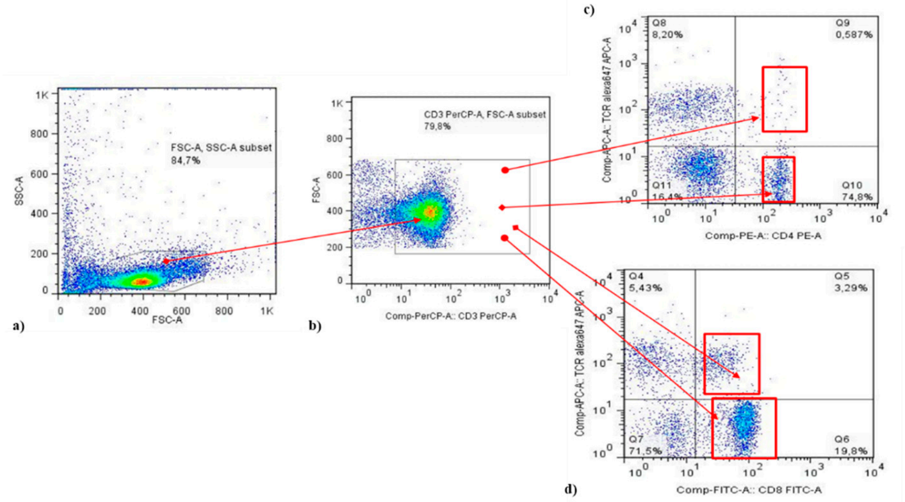

Figure S2. Gating strategy for T-panel. Representative gating strategy for T-cell subpopulations analysis. Mononuclear cells corresponding to lymphocytes were selected based on cell size and granularity (**Figure S2 a**) and T-cells identified as CD3^{+} (**Figure S2 b**). Subpopulations of T-cells were separated based on the expression of specific markers: TCR1^{+} for γδTCR subpopulation of T-lymphocytes and CD4^{+} for helper (-Th-) or CD8^{+} for cytotoxic T-cells (CTL). (**Figure S2 c & d**). The relative percentages of the αβTCR^{+} T-cell subpopulation was determined as CD3^{+}TCR1^{-} (non-γδ T-cells).
